# Medical Controversies

**Published:** 1917-10-06

**Authors:** 


### MEDICAL CONTROVERSIES.

Whether the war has sharpened our combative instincts generally, or whether it has merely brought certain questions to an acute issue, it is certain that its course has coincided with the development of a number of sharp medical controversies. There was in its early days much debate on the antiseptic dressing of wounds, and, apart from several minor points, this included a large question of principle in which some exceptionally heavy artillery was engaged. If the main cannonade has died down there are still lively local combats to be noted, as may be seen in the current discussion on the antiseptic action of "flavine." Some very ambitious announcements heralded the introduction of this substance, and the lay journalist was given the opportunity of presenting its virtues in a truly dramatic guise. Now we have a vigorous denial of these claims advanced by a highly experienced bacteriologist, who, however, finds the original sponsors by no means unskilled in defence and quite ready to carry the war into the enemy's country. Another animated discussion, and one pretending to wide issues, has been associated with heart disease. What is the clinical significance of cardiac murmurs might well have been regarded as an elementary question, yet the recruiting problem has provoked it into a heated debate, the echoes of which still linger in our ears. Schools of ardent advocates have been created by its influence, and both the "old" and the "new" have not wanted for doughty and skilful champions. Evidently the medical mind, in spite of its immersion in immediate practical work, is able to find time and energy for questions of the widest import, as well as art and enterprise in pressing the virtues of academic discussion.

Taking a general view of these controversies, we may offer a word of congratulation on the argumentative and dialectical skill and on the restraint and courtesy which the several controversialists display. If the "new" school may profess the attraction of enterprise and novelty, no evidence of senescence appears in the champions of the "old," and indeed one of these well merits the highest honours of the tourney. Persons apart, the contests show how difficult in the medical forum is the framing of large, comprehensive, and confident propositions. The generalisation to be secure must be founded on so extensive an experience, and must include due note of so many details, that none but minds of high competence can hope to compass it. Proverbially doctors differ, and a loose view puts down the differences to the ignorance and stupidity of the profession, whereas they really announce the complexity of the problems which medicine has to face. More encouraging is the intimation that freedom of thought and speech finds no restriction in the liberal atmosphere of the medical profession. Honest and sincere work, and doctrine issuing from it, are sure of an audience and a platform, and here is the best opportunity for truth to gain a recognition. Medicine professes but a limited knowledge and admits an extensive field where uncertainty and ignorance prevail. Controversy has its stimulating influence on the workers, and therefore it may be welcomed as an aid to the enterprise by which new truths are conquered.

